# Effects of a 12% carbohydrate beverage on tackling technique and running performance during rugby league activity: A randomised, placebo-controlled trial

**DOI:** 10.1371/journal.pone.0262443

**Published:** 2022-01-19

**Authors:** Nick Dobbin, Daniel Richardson, Liam Myler, Ozcan Esen

**Affiliations:** 1 Department of Health Professions, Manchester Metropolitan University, Manchester, United Kingdom; 2 Warrington Wolves Rugby League Club, The Halliwell Jones Stadium, Warrington, United Kingdom; University of Pavia: Universita degli Studi di Pavia, ITALY

## Abstract

The purpose of this study was to investigate the effects of a 12% carbohydrate (CHO) beverage on tackling technique and running performance during rugby league activity. Using a double-blind, placebo-controlled, randomised, crossover design, 15 academy rugby league players ingested a 250 ml bolus of a 12% CHO solution (30 g maltodextrin and 30 g sucrose in 500 ml) 15 minutes before two bouts of rugby activity. The rugby league match simulation for interchange players was used to standardise the movement patterns of activity and provide reliable outcome measures, whilst also reflecting the duration of a typical field-based conditioning session. Measures of tackling technique, external responses (e.g., fatigue index from sprint data) and rating of perceived exertion (RPE) were recorded throughout. Gut discomfort was measured before each bout. The interaction effect was largely compatible with the hypothesis for relative distance (*P*<0.001, *η*^2^ = 0.217) and fairly compatible for tackling technique (*P* = 0.068, *η*^2^ = 0.0640). The time effect for tackling technique, relative and high-intensity distance, sprint, and sprint to contact velocity, time at high metabolic power, PlayerLoad™, and RPE (all *P*<0.05; *η*^2^ = 0.131–0.701) was compatible with the hypothesis. Data for tackling technique, relative and high-intensity distance, sprint, and sprint to contact velocity, sprint, and sprint to contact fatigue index (all *P*<0.05; *η*^2^ = 0.189–0.612) was compatible with a supplement effect overall despite few differences in the pattern of change (interaction). Minimal gut discomfort was reported for the CHO (bout 1 = 27 ± 17; bout 2 = 23 ± 17 AU) and placebo (bout 1 = 23 ± 18 AU; bout 2 = 24 ± 13) trials. This study shows that a 12% CHO beverage before two bouts of standardised rugby activity is a practical and effective strategy for retaining tackling technique, increasing external responses, and reducing RPE without compromising gut comfort.

## Introduction

Rugby league requires players to perform frequent high-intensity actions (e.g., sprinting, accelerating, decelerating, and changing direction) that are interspersed with periods of low-intensity activity (e.g., walking, jogging or a ‘ball-out-of-play’ period) [1–3]. Rugby league also requires players to engage in high-impact collisions, wrestles and tackles, that when combined with high-intensity running, result in frequent repeated high-intensity efforts (RHIE) [4]. These high-intensity efforts have been associated with scoring and conceding a try [4], thus strategies that optimise athletes’ ability to execute each component of a RHIE are likely to be important for successful performance and potentially moderate injury risk [5]. Research indicates that contact events in rugby league (i.e., tackles and collisions) account for around for 61% of all injuries, with a significantly greater proportion occurring during the latter stages of a match [6]. In part, these findings may be explained by reductions in tackle technique as the session progresses, with clear evidence in other rugby codes demonstrating *moderate* to *large* differences in tackling technique between those who sustain an injury compared to those who do not [7]. Such observations may also be explained by fatigue that results in impaired tackle technique [8] and poor positioning before a tackle due to the observed decrements in relative and high-speed distance covered during a second half of a match [2]. As such, there is a need to consider potential strategies to maintain tackle technique as well as relative distance and high-speed distance during rugby league activity.

Various factors likely contribute to the impaired running performance (e.g., high-intensity distance) and tackling technique over the course of a rugby league training session or match-play. Of particular note is the metabolic cost of activity that is reported to be substantial [9–11], in part, due to the 11–30 contacts during training or 34–39 during match-play [12]. For example, the internal responses are higher when contact is included compared to when it is not [13], and a greater extent of muscle glycogen depletion is observed after match-play compared to simulated match-play [9, 14]. Furthermore, Dobbin et al. [15] recently reported a 2.1 to 8.9% drop in relative distance, high-speed distance, mean speed, and time at high metabolic power between two 23-minute bouts of rugby league activity. The same authors also reported that the fatigue index across sprints was 4.9 to 22% greater in the second half and that rating of perceived exertion was 12% higher [15]. As such, several research groups have explored various strategies of carbohydrate ingestion (alone or combined with other supplements) to maintain or improve running performance during single [14, 16, 17] or repeated [18] rugby activity. In brief, Bradley et al. [14] investigated the effects of high or low CHO intake in the 36 hours preceding a simulated rugby league match; Clarke et al. [16] investigated the effects of 6% CHO and caffeine on simulated rugby league match performance; and Hengist et al. [18] examined the effects of CHO ingestion in the 3 hours between two rugby sessions consisting of small-sided games. To date, only one study has included a CHO only trial that determined the effects of a 9% CHO beverage compared to a placebo and CHO with caffeine trial [17]. None of these studies provided any insight into the effects of CHO ingestion on other aspects of rugby such as tackling technique. Indeed, Roberts et al. [17] did include a motor skill task (pseudo hopscotch) within their study, demonstrating no effect of CHO, though the ecological validity of this test is limited. Furthermore, Roberts et al. [17] included a multiple dosing strategy whereby a large bolus (500 ml) was given 60 minutes before the activity with remaining solution given mid-way through the first half, at halftime and mid-way through the second half, which might be a challenge during a competitive match situation.

Research in other sports such as soccer have addressed several gaps highlighted previously, however extrapolating these findings to rugby is fraught with issues given the considerable differences in technical requirements when executing specific skills, the metabolic cost of the activity, and the participants’ characteristics. Nonetheless, the ingestion of CHO during exercise can result in exogenous CHO availability that may preserve central nervous system (CNS) integrity [19], reduce perceptions of effort during intermittent running [20] and support the retention of skill [21–24]. For example, Rodriguez-Giustiniani et al. [22] reported that the consumption of 2 x 250 ml CHO (12%; sucrose and maltodextrin) beverages before each half of a soccer simulation protocol resulted in greater total running capacity during a 20-meter shuttle test, and a higher passing score and accuracy during a passing task in the latter stages of their protocol. No difference in dribbling speed was observed. In contrast, Harper et al. [23] observed greater dribbling speed using university-standard soccer players in the final third of the protocol after the ingestion 2 x 250 ml of 12% CHO (sucrose, maltodextrin and isomaltulose). Whilst these finding support the notion that CHO ingestion, when given in two high concentration boluses, can be beneficial for improving external loads and skill performance, extrapolating these skills to rugby is not appropriate and research is required to explore if similar findings are evidence during rugby-specific activity.

In designing a study to test the hypothesis that CHO ingestion can improve the external loads (e.g. sprinting speeds, fatigue indices etc.) and skill performance in rugby, namely tacking technique, there are some key considerations that warrant discussion. Firstly, there is need to use a standardised measure of performance that is ecologically valid but also controls for large variation between playing position and matches and/or training sessions. As such, the use of a simulation protocol that fits within a typical field-based training session (~60 mins) and controls for this variability might provide a useful tool for assessing the efficacy of CHO ingestion on measures of rugby league performance. Secondly, supplements must have efficacy without compromising the health of an athlete and therefore, it is recommended to use a blend of multiple transportable CHO (e.g., maltodextrin and sucrose [25, 26]) to minimise gut discomfort [22, 27]. A blended approach is also reported to increase carbohydrate oxidation rate, thus facilitating players’ ability to perform RHIE [22, 28, 29]. Finally, the concentration is an important consideration, with research in rugby currently limited to 6–9% CHO. However, evidence from soccer suggests that a higher concentration (~12%) might have a more pronounced effect when CHO is ingested within close proximity (10–15 minutes) of the activity, may be a more feasible alternative to regular CHO feeding as this can be difficult to implement, and can reduce the risk of hypoglycaemia [30].

The aim of this study was to determine the effects of ingesting a 12% of CHO beverage on the tackling technique and running performance of rugby league players during simulated rugby activity. It was hypothesised that the consumption of a 250 ml CHO (12%) beverage before each bout would help retain tackling technique, increase the external responses, and result is a lower perceived exertion.

## Materials and methods

### Study design

**Fig 1:**
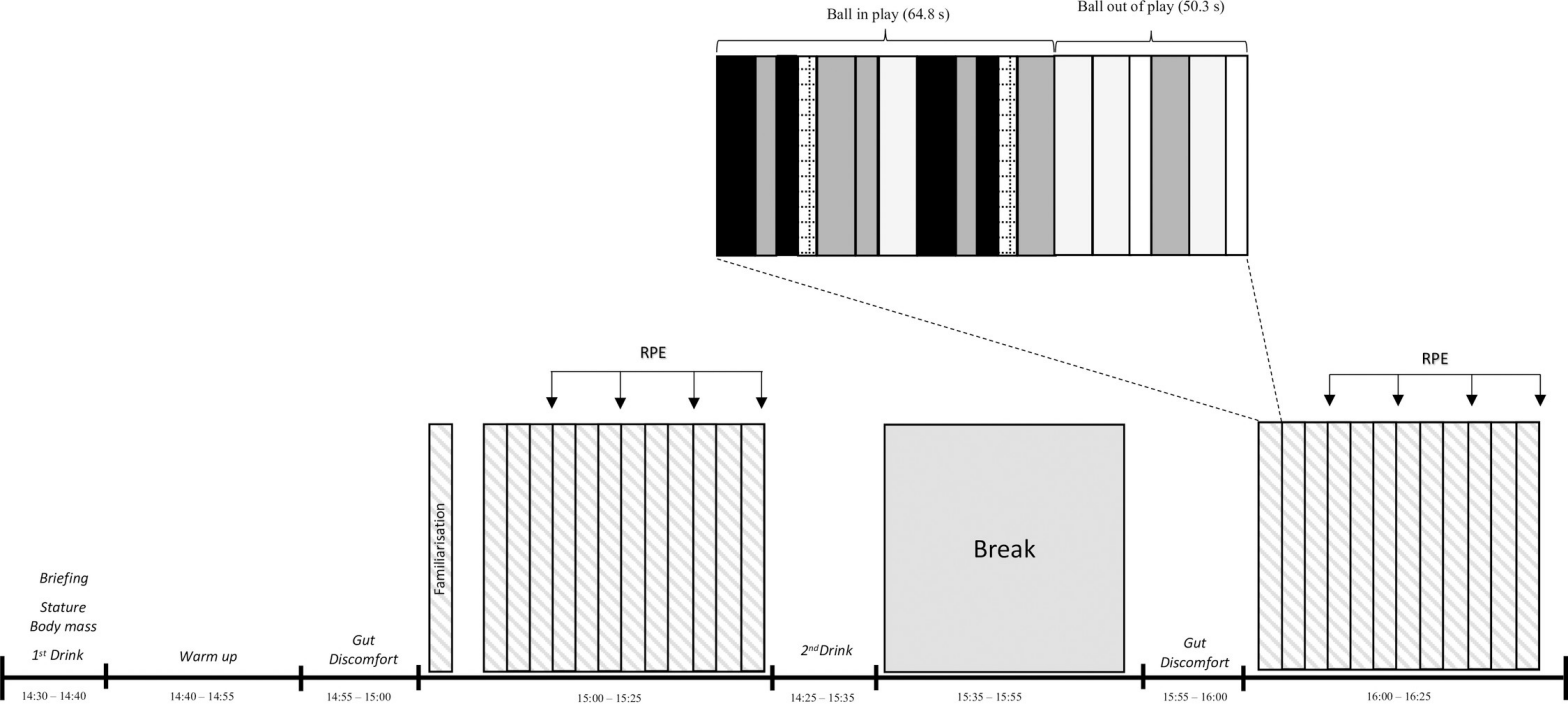
Schematic overview of the study design.

Using a placebo-controlled, double-blind, randomised crossover design, participants completed two trials of the rugby activity on an outdoor synthetic pitch, consuming either CHO or PLA 15 minutes before each bout (Fig 1). Participants were randomly allocated using an online number generator conducted by a non-involved research assistant. Both trials were performed at the same time of day (15:00h) separated by 48 hours of no activity to fit within the teams break in playing schedule. Environmental conditions were similar between trial 1 and 2 (temperature = 17 *cf*. 18°C, humidity = 85 *cf*. 81%, pressure 1004 *cf*. 1019 mbar). Participants were asked to record a 2-day food diary (15:00 to 15:00) before the first trial and repeated the same diet in the 2 days before the second trial, and avoid caffeine and any other ergogenic aid in the 24 hours before trials. The participants completed a countermovement jump on arrival and reported their wellbeing using a 1- to 5-point Likert scale for perceived fatigue, mood, muscle soreness, sleep quality, and stress. Participants wore the same playing kit and studded boots for both trials.

### Subjects

Eighteen academy rugby league players were recruited to participate in this study (mean ± SD: age = 17.8 ± 0.7 years, stature = 183.0 ± 4.7 cm; body mass = 87.0 ± 7.7 kg). All participants were contracted to a professional club, had a minimum of 3 years’ experience of rugby league and were free of injury. A sample size calculation was conducted *a-priori* where α was 0.05, power (1- β) was 0.8 and the effect size from the interaction effect (*η*^2^ = 0.365) for sprint speed was used [28], which indicated a minimum of 15 participants was required (G*Power 3.1.9.4, Universität, Düsseldorf). All participants provided written informed consent as they were all > 16 years and were competing in the academy Super League. All procedures were approved the Faculty of Health, Psychology and Social Care Research Ethics Committee, Manchester Metropolitan University and conducted in accordance with the Declaration of Helsinki.

### Beverage

The CHO beverage (2 x 250 ml) was a 12% solution that was a blend of maltodextrin (30 g, GI rating = 110) and sucrose (30 g, GI rating = 65) (Bulk Powders, UK) with an orange flavour. The PLA was taste- and texture-matched using sugar free cordial (Robinsons, Britivc PLC, UK) by a laboratory technician and checked by the lead researcher. Both contained similar amounts of sodium (20.5 mg per beverage). Fifteen minutes after consuming the drink and immediately before each bout of rugby activity, participants provided a rating for gut discomfort using a 0–100 visual analogue scale (0 = none– 100 = severe). At the end of the study, participants were asked about which trial they thought was the CHO trial.

### Procedures

To reliably assess tackle technique and running performance, we used the rugby league match simulation protocol for interchange players (RLMSP-i) [31], which encompasses all key actions performed in training and match-play. The RLMSP-i was not used with a view of assessing the effects of CHO ingestion for interchange players specifically, but to provide a reliable and standardised protocol that fitted within the club’s standard field-based conditioning time. Furthermore, previous research using this simulation has demonstrate a -0.4 to 8.9% drop in external loads, a 22% increase in the fatigue index and 12% increase in RPE [15]. The RLMSP-i comprised two 23-minute bouts, separated by a 20-min rest period. Participants responded to audio instructions that involved walking, jogging or sprinting between a series of cones (Fig 1). During each bout, 12 cycles of play were completed and included a ‘ball in-play’ period lasting 64.87 s and a ‘ball out of play’ period lasting 50.25 s. All players completed 1 full cycle to familiarise themselves with the protocol. Throughout the simulation players were instructed to perform each of the 48 sprints and 24 sprints to contact maximally (as the attacking player). The sprint to contact involved sprinting 8 m toward their partner and contacting the tackle shield (Centurion Rugby, West Yorkshire, UK) held by a teammate of similar stature (Δ4.6 ± 2.1 cm) and body mass (Δ2.2 ± 1.8 kg). The coefficient of variation (CV) of relative distance, high-speed running, peak speed and PlayerLoad™ during the RLMSP-i have been reported to be between 1.3 and 14.4% for rugby players [32].

At the point of contact during the protocol, participants were instructed to wrap their arms around the tackle shield and their opponent and attempt to turn 180° to gain dominance. After 3 seconds, the researcher called “held” and participants performed a “flapjack”. All tackles (*n* = 24) were recorded using a camera (37-mm digital video camera; DCR-TRV 950E; Sony, Nagasaki, Japan) affixed to a free-standing tripod in a sagittal plane and at a height of 90 cm (approx. waist height). Each tackle was analysed on a 6-point rugby league tackling technique criteria [33] using Kinovea software (Kinovea, V0.8.15, Kinovea org., France). The criteria included: a) contacting the target in the centre of gravity, (b) contacting the target with the shoulder, (c) body position squared and aligned, (d) leg drive upon contact, (e) watching the target onto the shoulder, and (f) centre of gravity forward of base of support. Players were awarded 1 point for each of the criteria they met. Application of the tackling criteria was completed by a single researcher (~ 3 years’ experience in rugby league) with a selection (*n* = 6 per player) re-analysed by a second researcher (6 years’ experience in rugby league) with an interclass correlation of 0.97. The grading of tackle technique has been validated in previous rugby league research and the CV is reported to be 3.3% [33].

### External and perceptual responses

The external responses were recorded using a 10 Hz microtechnology device (S5 Catapult Innovations, Melbourne, Australia) fitted into a custom-made vest with the device positioned between the participant’s scapulae. The mean ± SD number of satellites and horizontal dilution of precision was 12 ± 0 and 0.9 ± 0.1. Relative and high-speed (> 3.9 m·s^-1^) distance, peak speed of all sprints, and sprint to contact speed for the attacking participant were determined and averaged for each quarter. Using the peak sprint speed (*n* = 48) and sprint to contact speed (*n* = 24), a fatigue index (FI) was calculated using the following equation: FI = 100*EXP(slope/100), where the slope is calculated using the line of best fit for 100*natural logarithm of sprint data*(number of sprints-1) [34]. The built-in 100-Hz triaxial accelerometer, gyroscope, and magnetometer were used to determine time at high metabolic power (HMP; >20 W·kg^-1^) and PlayerLoad™. Data was downloaded using OpenField Cloud (Catapult Sport, Melbourne, Australia), and truncated for each quarter, individual sprints and sprints to contact. Rating of perceived exertion (RPE) was recorded at the end of each quarter using the Borg [35] 6–20 scale (CV = 11.2–13.7%).

### Statistical analysis

Data are presented as mean ± standard deviation (SD). Normality and homogeneity of variance were checked using the Shapiro-Wilk and Levene test, respectively, confirming a normal distribution. Data analysis across quarters was averaged. A two-way ANOVA was used to firstly determine if an interaction effect between supplement and time existed before examining the time and supplement effect using to collapsed means. Where a significant time-effect was apparent, post-hoc analysis was carried out with Bonferroni adjustment. The effect size for variance analysis was determined using the partial eta squared statistic (*η*_p_^2^) with values of ≥ 0.01, ≥ 0.06 and ≥ 0.14 indicating small, moderate, and large effects, respectively [36]. Comparisons in nutrition intake, wellbeing and CMJ flight time were supplemented with a standardised mean difference (SMD) with 95% confidence limits. SMDs were interpreted as < 0.20, trivial; 0.21–0.60, small; 0.61–1.20, moderate; 1.21–2.00, large; > 2.00, very large [37]. Statistical analysis was performed using SPSS for Mac (Version 26, IBM, Armonk, NY, United States) with *P*-values presented as absolute values or *P* < 0.001 where appropriate and interpreted on a scale of completely incompatible (P = 1.0) to compatible (P < 0.001) with the hypothesis.

## Results

Fifteen participants completed both trials and all outcome measures. From the initial sample (*n* = 18), three participants had trained with the senior team on the day of the second trial as so were excluded due to the potential influence this would have on their performance relative to trial 1. The difference in dietary intake between trials 1 and 2 for CHO (3.5 ± 0.7 *cf*. 3.6 ± 0.7 g·kg^-1^; SMD = 0.21 ± 0.58, *P* = 0.155), protein (2.5 ± 0.4 *cf*. 2.6 ± 0.3 g·kg^-1^; SMD = 0.26 ± 0.60, *P* = 0.189), fat (1.1 ± 0.2 *cf*. 1.1 ± 0.2 g·kg^-1^; SMD = 0.04 ± 0.52, *P* = 0.776) and total energy intake (2768 ± 361 *cf*. 2831 ± 393 kcal; SMD = 0.17 ± 0.57, *P* = 0.257) were considered trivial to small. Furthermore, analysis of dietary intake also revealed that the timing of CHO, protein and fat was similar between trials, with the final meal consumed 180 ± 30 minutes before arrival. Minimal difference was observed in pre-trial countermovement jump flight time (0.55 ± 0.04 *cf*. 0.54 ± 0.04; SMD = 0.04 ± 0.51, *P* = 0.374). A trivial difference was observed for total wellbeing between trial 1 (17.2 ± 2.2 AU) and 2 (16.8 ± 1.2 AU) (SMD = -0.17 ± 0.38, *P* = 0.31).

**Fig 2:**
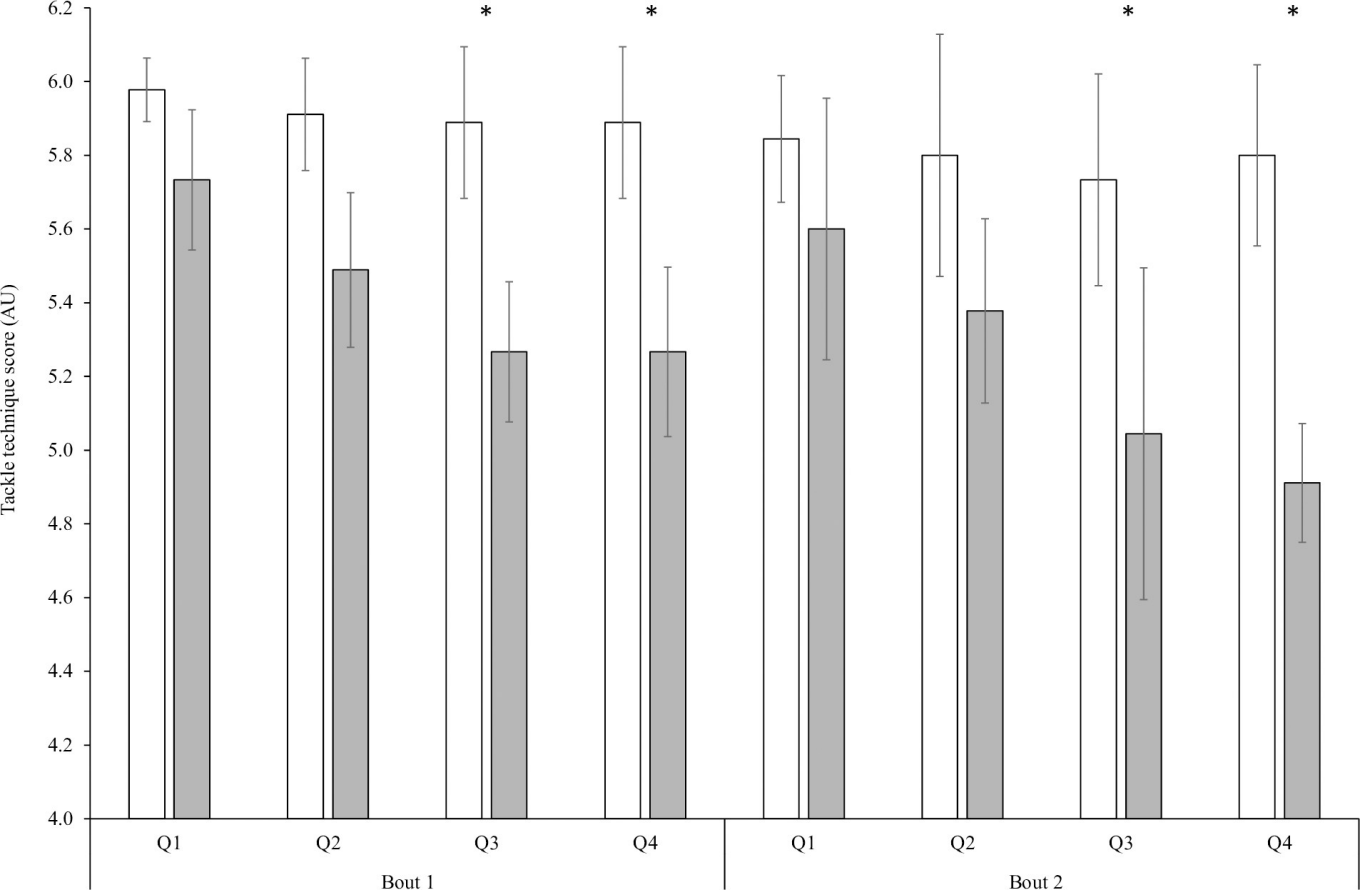
**Mean (SD) tackle technique score for CHO (white bars) and placebo (grey bars) across quarters of the RLMSP-i.** * indicates significantly difference to Q1 in the first bout.

The interaction effect between supplement and time observed for tackling technique was fairly compatible with the hypothesis (F = 1.919, *P* = 0.068, *η*_p_^2^ = 0.064), though the type one error was slightly above the conventional 5% (Fig 2). The ingestion of CHO resulted in a higher mean tackling technique score when compared to PLA (F = 44.176, *P*<0.001, *η*_p_^2^ = 0.612). A main effect of time was also observed for tackling technique, with comparisons between quarters shown in Fig 2 (F = 4.208, *P* = 0.007, *η*_p_^2^ = 0.131).

**Fig 3:**
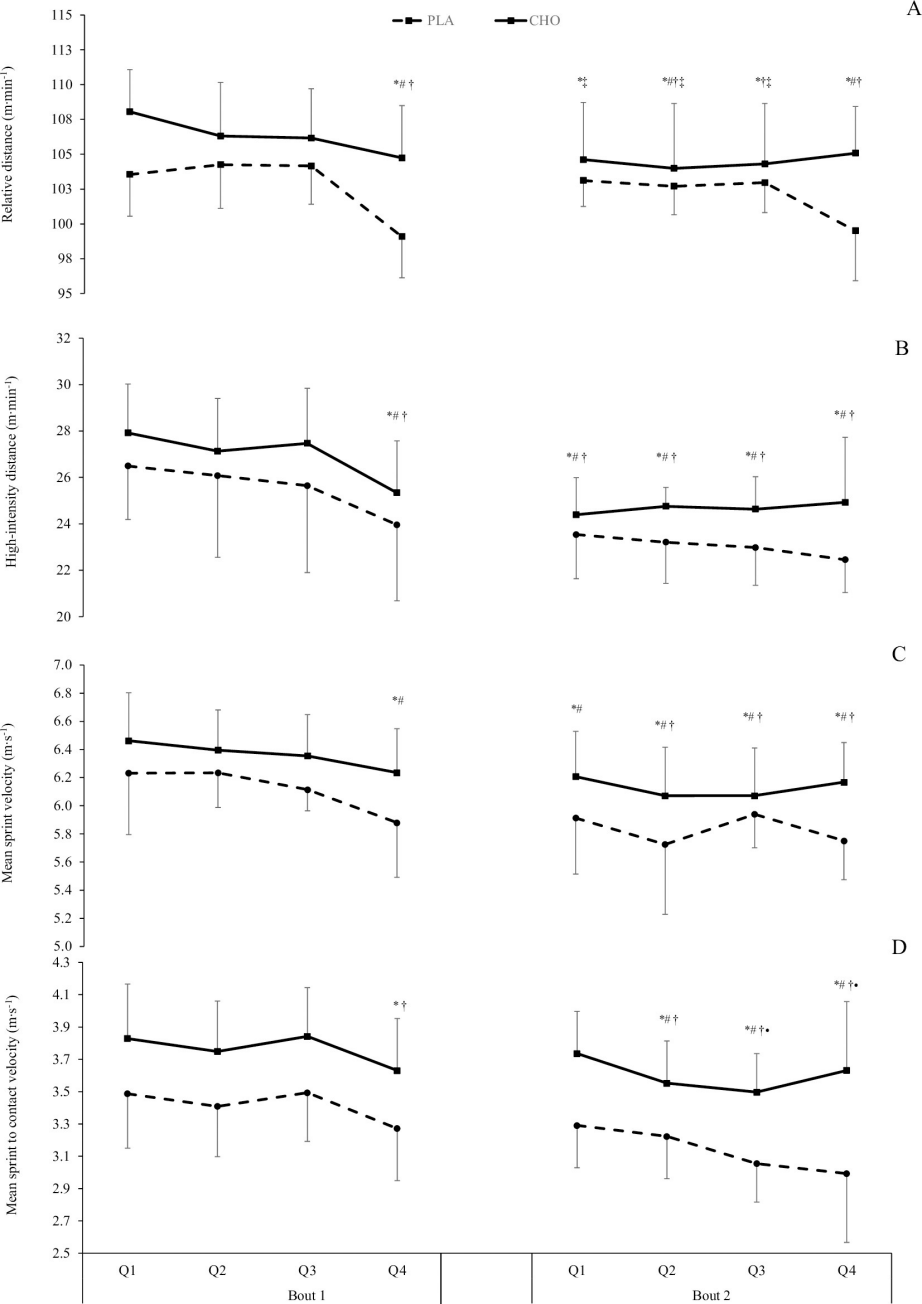
Mean (± SD) relative distance (A), high-intensity distance (B), sprint velocity © and sprint to contact velocity (D) with CHO (solid line) and placebo (dashed line) at each quarter of the RLMSP-i. *, #, †, ‡ and • indicate a time-effect compared to Q1, Q2, Q3, Q4 of the first half and Q1 second half, respectively.

The interaction effect between supplement and time was compatible with the hypothesis for relative distance (F = 7.761, *P* <0.001, *η*_p_^2^ = 0.217), but not relative high-intensity distance (F = 0.577, *P* = 0.774, *η*_p_^2^ = 0.020), sprint velocity (F = 1.114, *P* = 0.356, *η*_p_^2^ = 0.038) or sprint to contact velocity (F = 2.064, *P* = 0.490, *η*_p_^2^ = 0.069) (Fig 3). A negative effect of time was observed across the simulation for relative distance (F = 16.874, *P*<0.001, η_p_^2^ = 0.376), relative high-intensity distance (F = 20.617, *P*<0.001, *η*_p_^2^ = 0.424), mean sprint velocity (F = 13.115, *P*<0.001, η_p_^2^ = 0.319) and sprint to contact speed (F = 16.882, *P*<0.001, *η*_p_^2^ = 0.376). The supplement effect indicated that after ingesting CHO, a higher relative distance (F = 8.251, *P* = 0.008, *η*_p_^2^ = 0.228), relative high-intensity distance (F = 6.540, *P* = 0.016, *η*_p_^2^ = 0.189), mean sprint velocity (F = 10.617, *P* = 0.003, *η*_p_^2^ = 0.275) and sprint to contact speed (F = 30.621, *P*<0.001, *η*_p_^2^ = 0.522), though the pattern of change compared the PLA across timepoints was similar.

**Fig 4:**
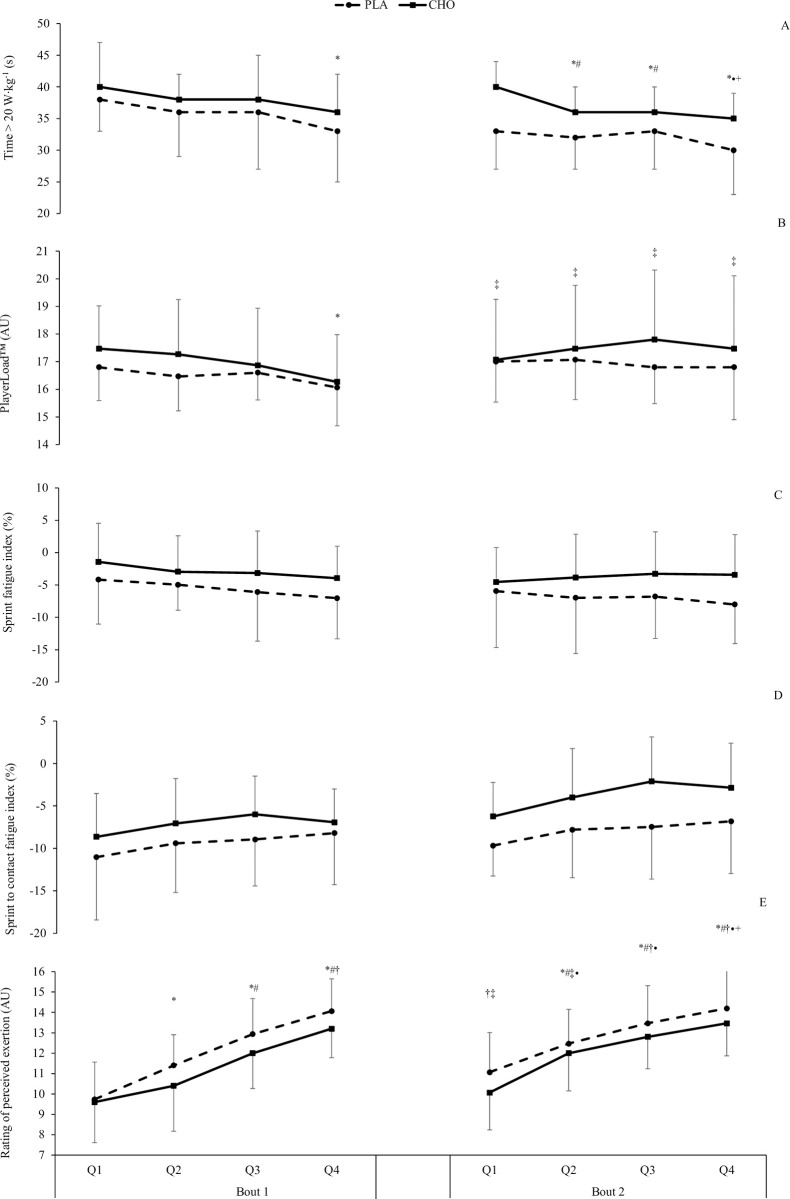
Mean (± SD) HMP time (A), PlayerLoadTM (B), sprint fatigue index (C), sprint to contact fatigue index (D) and rating of perceived exertion (E) with CHO (solid line) and placebo (dashed line) at each quarter of the RLMSP-i. *, #, †, ‡ indicate significant difference compared to Q1, Q2, Q3 and Q4 of the first half respectively. • and + indicate a significant difference compared to Q1 and Q3 of the second half, respectively.

The interaction effect for HMP (F = 1.588, *P* = 1.410, *η*_p_^2^ = 0.054), PlayerLoad™ (F = 0.693, *P* = 0.678, *η*_p_^2^ = 0.024), sprint FI (F = 1.168, *P* = 0.323, *η*_p_^2^ = 0.040), sprint to contact FI (F = 0.044, *P* = 0.874, *η*_p_^2^ = 0.016) or RPE (F = 1.649, *P* = 0.124, *η*_p_^2^ = 0.056) was mostly incompatible with the hypothesis. A negative effect of time was observed for HMP (F = 8.660, *P*<0.001, *η*_p_^2^ = 0.236), PlayerLoad™ (F = 3.626, *P* = 0.009, *η*_p_^2^ = 0.115) and RPE (F = 65.537, *P*<0.001, *η*_p_^2^ = 0.701) but not sprint FI (F = 1.705, *P* = 0.110, *η*_p_^2^ = 0.057) or sprint to contact FI (F = 1.205, *P* = 0.302, η_p_^2^ = 0.041) (Fig 4). CHO resulted in a reduced sprint (F = 25.744, *P*<0.001, η_p_^2^ = 0.479) and sprint to contact (F = 7.385, *P* = 0.011, η_p_^2^ = 0.209) FI compared to the PLA trial, though demonstrated a similar pattern of change across quarters.

No interaction (F = 1.080, *P* = 0.308, η_p_^2^ = 0.037), supplement (F = 4.086, *P* = 0.772, η_p_^2^ = 0.003) or time effect (F = 0.179, *P* = 0.675, η_p_^2^ = 0.006) was observed for gut discomfort. The mean value for the CHO condition was 27 ± 17 AU (1^st^ bout) and 23 ± 17 AU (2^nd^ bout). For the PLA condition, the mean value was 23 ± 18 AU (1^st^ bout) and 24 ± 13 AU (2^nd^ bout). A total of 6 players (40%) of players correctly identified the CHO trial whereas the remaining players were incorrect.

## Discussion

This study assessed the effects of a multiple source, high-concentration CHO beverage on tackling and running performance in academy rugby league players. The results provide evidence to suggest that CHO ingested immediately before two bouts of simulated rugby league match activity resulted in higher tackling technique and external responses for a given RPE from the outset of the activity despite observing a similar change over the course of the intervention compared to the PLA trial. The results for relative distance and tackling technique indicated CHO supported the maintenance of these variable. There was no difference in gut discomfort between CHO and PLA conditions nor was there any difference in wellbeing or countermovement jump flight time, suggesting residual fatigue was unlikely to alter the results of this study.

The results of this study suggest that ingesting a highly concentrated CHO beverage may attenuate the observed reduction in tackling technique over the course of 2 x 23 minutes period of rugby league during the PLA condition. Whilst no interaction effect was observed at a level of 0.05, our results indicate that tackling technique remained largely constant across the bouts when ingesting CHO. Further, the probability of this finding being a type one error was only slightly above the conventional 5% (6.8%), and so given a type 1 error is likely to overly detrimental, this finding might be practically meaningful. Indeed, the percentage change between the first and last three tackles during the protocol was -2.9% for the CHO condition, which is below the CV (3.3%; [33]) for this assessment. In contrast, the equivalent change for the PLA condition was -14.3%. The finding that tackling performance was attenuated agrees with previous literature reporting the benefits of CHO for retaining skill in soccer including aspects of dribbling [28], shooting [23] and passing performance [22]. However, we highlight that in previous literature, the effects of CHO ingestion were only evident within the last 15 to 30 minutes of the protocol whereas in this study, differences between CHO and PLA were evident at all timepoints. Such findings may be explained by the difference skills being assesses, the demands of performing these skills, and the frequency this was measured throughout the protocol. The observation that tackle technique was slightly higher in Q1 reflects the higher sprint and sprint to contact speed that allowed for improve positioning and leg drive. The effect of CHO on skill performance in previous research has been attributed to greater preservation of the CNS and improved motor control [22, 23] despite baseline glucose values above that consider for hypoglycaemia (~ 4 mmol/L). Whilst such mechanisms might explain our results, though we cannot confirm, it is also worth noting that the change in tackle technique was largely attributed to the reduced scores for leg drive criteria and being square and aligned to the opponent. The reduction in scores for the leg drive criteria during the PLA condition may be caused by a lower sprint to contact velocity, higher FI and higher RPE, thus less running momentum and force. The lack of ability to be squared and aligned may be the result of impaired sprint speed and the resulting reduction in preparation time. However, further research that uses a placebo and non-placebo trial is required to confirm this as well as determine the magnitude of the effect. Such findings might have important implications for tackle success in rugby given their frequency and importance on the overall outcome of a match [4] as well as the associated injury risk with poor tackle technique [7].

Participants demonstrated a higher relative and high-intensity distance, and sprint and sprint to contact velocity across the two bouts of activity when ingesting CHO, albeit the pattern of change across time was similar with the PLA trial. Similarly, HMP and PlayerLoad™ were higher in the CHO condition, suggesting a greater ability or willingness to perform metabolically demanding actions, which may link back to the leg-drive criterion. Interestingly, all external responses were higher in the CHO condition from the outset of the activity, suggesting an altered the pacing profile adopted by participants [16], whereby this greater load was perceived to be manageable for the duration of the activity. Thus, despite few showing an interaction effect, the overall supplement effect might be practically important. These findings are likely explained by several factors. Firstly, whilst the activity used in this study was well-structured with a consistent duration and distance, there is scope for participants to cover greater distance during the deceleration phase following a maximal sprint as well as distance travelled during the contact. As such, it is possible that, during the CHO condition, where participants displayed greater sprint velocity, the distance required to decelerate was greater. Similarly, due to the higher sprint to contact velocity and increased running momentum, the distance covered in the collision was potentially increased for the attackers (forwards) and defender (backwards). Secondly, the greater external responses reported across quarters may be explained by using multiple transportable CHO that spares muscle glycogen via greater liver glycogen repletion [11, 22, 38]. Finally, the ingestion of CHO is suggested to alter the perception of effort during prolonged high-intensity intermittent exercise [16, 28] along with perceptions of activation (e.g. readiness to perform an activity) and pleasure/displeasure [23, 28]. The pacing profile exhibited during this study is closely linked to RPE where this is often used to regulate the intensity of exercise [16]. In this study, we observed a lower RPE across all bouts of activity despite higher external loads, which may be explained by a sensory benefit where CHO activates areas of the brain associated with perceived exertion and reward via the oral receptors [39].

Whilst this study offers new and important insight, there are several limitations worthy of comment. Firstly, the use of a shortened rugby league simulation was used in this study to control for variability between training session and matches whilst also fitting within the club’s current training schedule. We do acknowledge that duration of activity plays an essential role in CHO oxidation and muscle glycogen utilisation, though our findings suggest that CHO ingestion may be beneficial during short (< 60 mins) but intense activity such as that used in this study. Secondly, due to the data being collected on all participants simultaneously with limited equipment and personnel, we were unable to measure blood glucose concentration within a timeframe that would allow for accurate interpretation. Whilst blood glucose would have supported our interpretation, numerous studies exist reporting the changes in glucose following ingestion have demonstrated that changes in performance do not appear to be directly associated with blood glucose concentration [22, 23, 30]. Thirdly, we were unable to include a control trial within this study due to the logistics of conducting research during a competitive season. However, this requires acknowledgment as the magnitude of difference between placebo and CHO is likely to be smaller than a control and CHO trial. Finally, we used a validated method of scoring tackling technique that was only concerned with the tackle itself and could not quantify the speed of detection of an opponent, the ability to react to the opposition’s movement or team structure, or assess the magnitude of the collision.

In conclusion, the consumption of a 12% CHO beverage before multiple rugby-specific activities (e.g., conditioning, skills, game-based training) may be an effective nutritional strategy for retaining tackling technique, increasing external responses, and reducing the perceptual response for a given external workload from the outset of activity. Further, given the lack of interaction effect that suggest the pattern of response is similar to PLA, starting from a higher position (e.g. higher tacking technique score) might be practically meaningful. This study provides evidence that a single 250 ml bolus of 12% CHO given immediately before a bout of activity is a practical and effective strategy. Furthermore, practitioners working in rugby can apply the findings of this study with the knowledge that it causes no greater gut discomfort that the PLA condition and provides an ergogenic effect to the individual.
